# Ceftriaxone-induced severe hemolytic anemia, renal calculi, and cholecystolithiasis in a 3-year-old child: a case report and literature review

**DOI:** 10.3389/fphar.2024.1362668

**Published:** 2024-03-15

**Authors:** Enfu Tao, Huangjia Zhou, Meili Zheng, Yisha Zhao, Junfen Zhou, Junhui Yuan, Tianming Yuan, Changhua Zheng

**Affiliations:** ^1^ Department of Neonatology and NICU, Wenling Maternal and Child Healthcare Hospital, Wenling, Zhejiang Province, China; ^2^ Department of Pediatrics, Wenling Maternal and Child Healthcare Hospital, Wenling, Zhejiang Province, China; ^3^ Department of Neonatology, Children’s Hospital, Zhejiang University School of Medicine, National Clinical Research Center for Child Health, National Children’s Regional Medical Center, Hangzhou, Zhejiang Province, China

**Keywords:** ceftriaxone, hemolytic anemia, hemolytic crisis, adverse drug reaction, renal calculi, cholecystolithiasis

## Abstract

Ceftriaxone is widely used in pediatric outpatient care for its efficacy against respiratory and digestive system infections, yet its increasing association with severe immune hemolytic reactions requires heightened vigilance from pediatricians. This report details a rare and severe case of ceftriaxone-induced severe immune hemolytic anemia (IHA), hemolytic crisis, myocardial injury, liver injury, renal calculi, and cholecystolithiasis in a previously healthy 3-year-old child. The child, treated for bronchitis, experienced sudden pallor, limb stiffness, and altered consciousness following the fifth day of ceftriaxone infusion, with hemoglobin (Hb) levels precipitously dropping to 21 g/L. Immediate cessation of ceftriaxone and the administration of oxygen therapy, blood transfusion, intravenous immunoglobulin (IVIG), and corticosteroids led to a gradual recovery. Despite initial improvements, the patient’s condition necessitated extensive hospital care due to complications including myocardial injury, liver injury, renal calculi, and cholecystolithiasis. After a 12-day hospital stay and a 3-month follow-up, the child showed complete normalization of Hb and liver function and resolution of calculi. In children, ceftriaxone infusion may trigger severe, potentially fatal, hemolytic reactions. Pediatricians must promptly recognize symptoms such as pallor, limb stiffness, and unresponsiveness, indicative of ceftriaxone-induced severe IHA, and immediately discontinue the drug. Effective management includes timely blood transfusion, respiratory support, IVIG administration, and corticosteroids when necessary, along with rigorous vital signs monitoring. Continued vigilance is imperative, even after cessation of ceftriaxone, to promptly address any residual adverse effects.

## Introduction

Ceftriaxone is a very common β-lactam antibiotic used to treat infectious diseases in children, with a broad spectrum against many infections. It is also the most commonly used antibiotic for respiratory and digestive system infections in pediatric outpatient settings (Hodgson et al., 2022). However, with the increasing use of ceftriaxone in pediatric medicine, a growing number of adverse drug reactions (ADRs) have been reported (Cai et al., 2021). The majority of adverse reactions caused by ceftriaxone are relatively mild, such as gastrointestinal hepatobiliary disorders (Zeng et al., 2020). Less common ADRs include kidney injury (Zhang et al., 2023a), ceftriaxone-induced cholestatic hepatitis (Castellazzi et al., 2022; Eldougdoug et al., 2023), hemolytic anemia (Northrop and Agarwal, 2015), and lithiasis (Louta et al., 2023). However, in very rare cases, it can lead to serious ADRs, such as hemolysis crisis, renal failure, multiple organ failure (Bell et al., 2005) and even death (Van Buren et al., 2018)**.**

Autoimmune hemolytic anemia (AIHA) is characterized by the heightened destruction of red blood cells (RBC), typically triggered by autoantibodies targeting antigens present on the surface of erythrocytes (Berentsen and Barcellini, 2021). AIHA is a rare condition in children, with an estimated incidence ranging from 1 to 3 cases per 100,000 people per year (Kar et al., 2024). AIHA displays a multifactorial pathogenesis, including genetic factors, infections, autoimmune diseases, and medications, etc (Fattizzo and Barcellini, 2022). In studies involving patients with autoimmune hemolytic anemia (AIHA), drug-induced immune hemolytic anemia (DIIHA) constitutes approximately 10% of the total cases (Hill et al., 2017). More than 130 drugs are suspected to trigger immune hemolytic anemia (IHA) (Maquet et al., 2024), with ceftriaxone being the 11th most common cause in children, occurring at a rate of approximately 1.5 cases per million in population (Tang et al., 2023). Compared to the rare incidence of ceftriaxone-induced IHA, ceftriaxone-induced renal calculi and cholecystolithiasis are relatively more common. The reported incidence of ceftriaxone-induced renal calculi ranges from 0.6% to 7.8% across different studies (Wang et al., 2017). Furthermore, ceftriaxone-induced cholecystolithiasis is even more prevalent, with an incidence of up to 43.10% in children treated with ceftriaxone (Meng et al., 2010).

DIIHA are rare and difficult to diagnose. Knowledge and clinical suspicion are fundamental to identify the phenomenon (Barcellini and Fattizzo, 2023). Diagnosis is typically achieved by integrating clinical history with the manifestations of hemolytic anemia (Michel, 2011; Wang et al., 2017). It primarily relies on identifying anti- RBC autoantibodies through the direct antiglobulin test (DAT), and secondarily on ruling out other potential causes of hemolytic anemia (Michel, 2011). Additionaly, ultrasound examination can help diagnosis ceftriaxone-induced renal calculi and cholecystolithiasis (Meng et al., 2010; Gökçe et al., 2014; Serdaroglu et al., 2016; Wang et al., 2017). If DIIHA is suspected, relevant medication should be stopped (Hill et al., 2017). In acute and severe cases, blood transfusions may be necessary, along with admission to intensive care (Barcellini and Fattizzo, 2023). Moreover, the decision to initiate corticosteroid therapy must be individualized and will depend on the severity of hemolysis and the degree of clinical suspicion that hemolysis is drug-induced (Hill et al., 2017). Conservative management is favored for asymptomatic ceftriaxone-induced renal calculi and cholecystolithiasis, as they often resolve spontaneously without surgical intervention (Meng et al., 2010; Gökçe et al., 2014; Wang et al., 2017).

We present a rare case of a previously healthy 3-year-old child who, on the fifth day of continuous ceftriaxone infusion, suddenly developed severe IHA, hemolytic crisis, myocardial injury, liver injury, renal calculi, and cholecystolithiasis. With prompt medical intervention, the child’s condition was successfully stabilized, averting a life-threatening situation.

## Case description

A previous healthy 3-year-old boy developed a cough without an apparent cause a week ago and sought medical attention at our pediatric outpatient clinic. Bronchitis was considered as the diagnosis, and the patient received a 4-day treatment of intravenous ceftriaxone in our outpatient setting. On the fifth day, during the intravenous administration of ceftriaxone, the child suddenly exhibited pallor, limb stiffness, and unresponsiveness. Physical examination revealed that the patient presented with a pale complexion. Pupils were responsive to light. Vital signs indicated a temperature of 36°C, a pulse rate of 160 beats/min, a respiratory rate of 20 breaths/min, and a blood pressure of 100/70 mmHg. Coarse breath sounds were noted in both lungs without the presence of rales. The heart rhythm was regular with normal heart sounds. Abdominal palpation revealed softness, and there were no neurological abnormalities. No rash was observed throughout the body, and the capillary refill time was less than 2 s. ADRs of ceftriaxone was suspected. The ceftriaxone infusion was immediately discontinued and the child was transferred to the pediatric emergency department, where oxygen therapy through a nasal cannula led to an improvement in skin color. Vital signs of the patient were closely monitored and oxygen saturation was displayed as 100%. Emergency complete blood count (CBC) revealed a hemoglobin (Hb) of 42 g/L, which was dramatically lower the normal range. Emergency blood biochemistry showed significant blood glucose elevation with 15.4 mmol/L, which was significantly above the normal range. Liver and kidney functions, as well as electrolytes, showed no obvious abnormalities. After half an hour, the child again displayed pallor, slight gaze fixation, increased muscle tone, and a decrease in heart rate. Immediate treatment included oxygen administration through a face mask, intravenous adrenaline for cardiac support, intramuscular midazolam for convulsion control, and intravenous methylprednisolone for anti-inflammation. Emergency second CBC revealed a Hb declined to 21 g/L. An emergency head and whole thoracoabdominal computed tomography were performed and no obvious bleeding in important organs such as the brain, lungs, liver, spleen, and gastrointestinal tract were observed. The emergency blood gas analysis showed a pH of 7.02, actual bicarbonate of 5.5 mmol/L, base excess of −24.03 mmol/L and lactic acid of 18 mmol/L, indicating severe metabolic acidosis and significant tissue hypoxia, and also suggesting that the child urgently needs red blood cell (RBC) supplementation to alleviate tissue hypoxia. Therefore, after cross-matching, a transfusion of 200 mL concentrated RBC with blood type O, Rh-positive, was administered promptly. Coagulation function shows no obvious abnormalities. In addition, intravenous immunoglobulin (IVIG) was administered to control immune hemolysis. After more than 1 hour, reexamination of blood gas showed a pH of 7.24, an actual bicarbonate of 11.8 mmol/L, base excess of −14 mmol/L and lactic acid of 16.3 mmol/L, indicating metabolic acidosis despite showing some improvement compared to the previous condition. Accordingly, acidosis was corrected with sodium bicarbonate. In addition, lactic acid seemed to decrease, suggesting tissue hypoxia began to improve. Reexamination of CBC revealed white blood cell (WBC) of 39.3 × 10^9^/L, Hb 46 g/L, N% 55.2%, suggested Hb was elevated after transfusion, and WBC was dramatically increased, which was considered due to stress reaction. The emergency electrolyte and renal function revealed no apparent abnormalities. The child exhibited notable improvement following interventions including oxygen therapy, blood transfusion, intravenous administration of IVIG, and correction of acidosis. Subsequently, he was transferred to the pediatric ward for advanced medical management.

**FIGURE 1:**
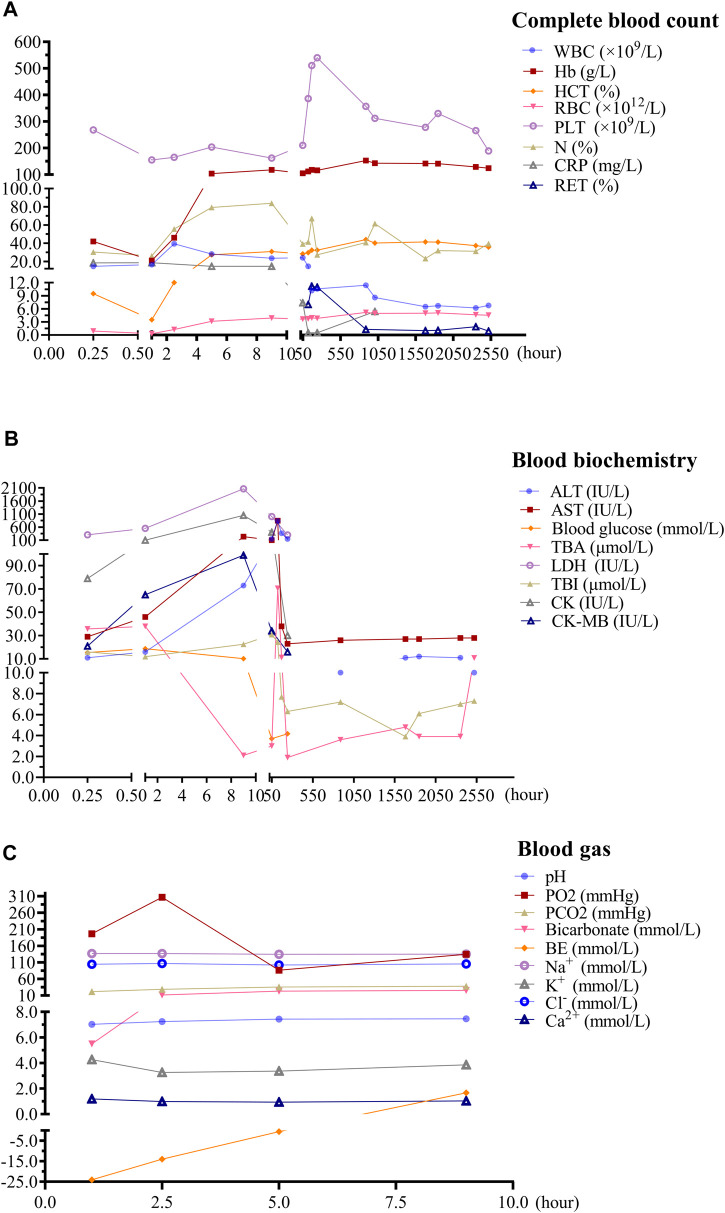
Temporal changes in laboratory tests following ceftriaxone-induced immune hemolysis. **(A)**:Temporal changes in complete blood cell following ceftriaxone-induced immune hemolysis; **(B)**:Temporal changes in blood biochemistry following ceftriaxone-induced immune hemolysis; **(C)**:Temporal changes in arterial blood gas following ceftriaxone-induced immune hemolysis. Outpatient blood indices were re-examined on the second, fifth, seventh, 10th, 37th, 42nd, 70th, 77th, 98th, and 105th days after discharge, respectively. WBC: white blood cell, RBC: red blood cell, Hb: hemoglobin, HCT: hematocrit, PLT: platelet count, N: neutrophil, CRP: C-reactive protein, RET: reticulocyte, ALT: alanine aminotransferase, AST: aspartate aminotransferase, TBI: total bilirubin; TBA: total bile acid, LDH: lactate dehydrogenase; CK: creatine kinase, CK-MB: creatine kinase-MB isoenzyme. pH: potential of hydrogen, PO2: partial pressure of oxygen, PCO2: partial pressure of carbon dioxide, BE: base excess.

**FIGURE 2:**
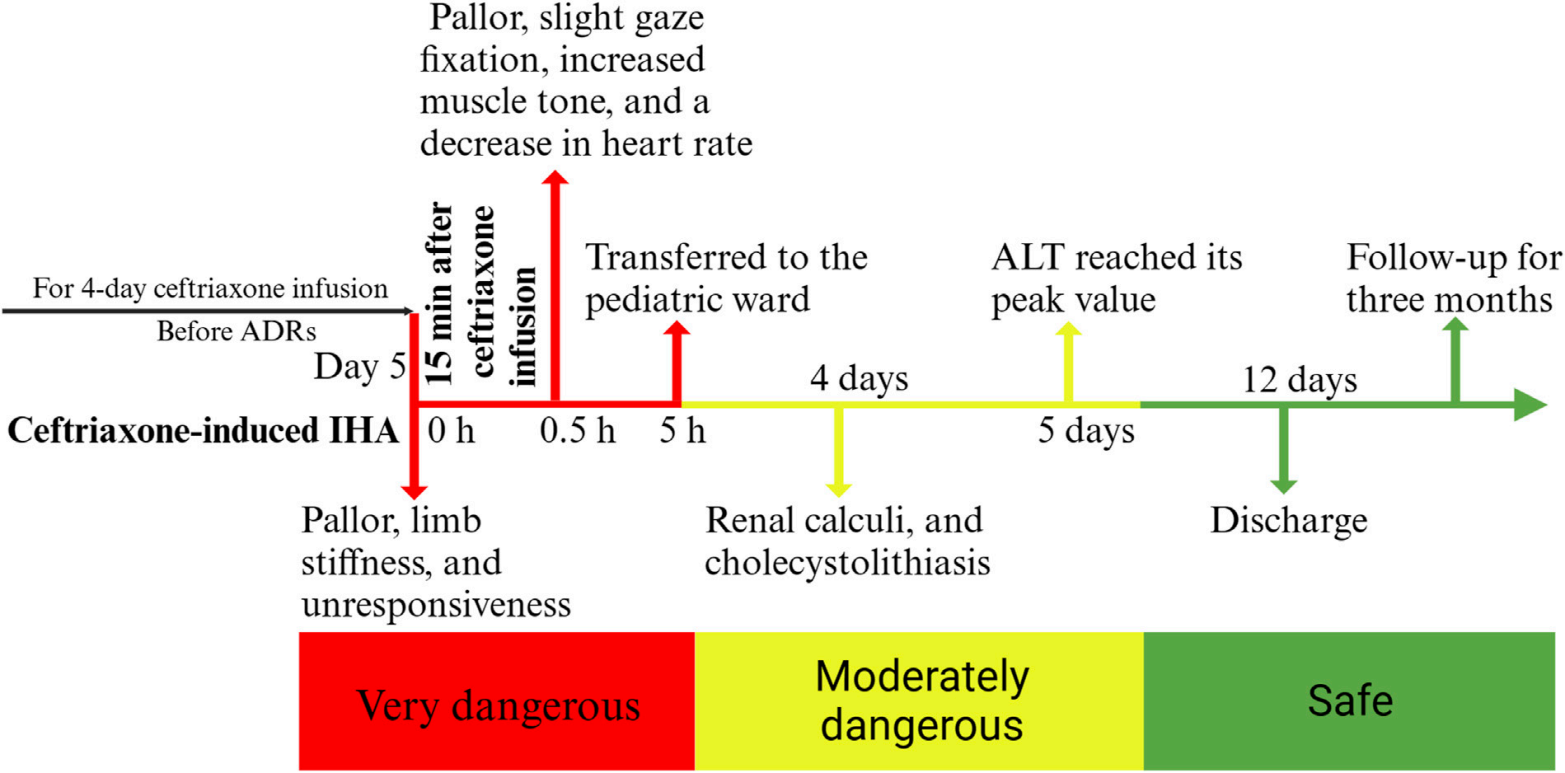
A timeline of the child’s progress from the onset of ceftriaxone-induced immune hemolysis to recovery, discharge, and follow-up. ADRs: adverse drug reactions, ALT: alanine aminotransferase.

During the hospitalization, the child continued to receive methylprednisolone for anti-inflammatory treatment for 3 days, then was switched to oral prednisolone acetate for 4 days with a gradual reduction in dosage and eventual discontinuation. Hemolysis screening indicated a positive direct antiglobulin test (DAT), supporting immune hemolysis induced by ceftriaxone. The child’s Hb levels quickly recovered, but blood biochemistry showed a significant increase in alanine transaminase (ALT), indicating liver injury, which peaked on the fifth day of hospitalization. Liver protection treatment with compound glycyrrhizin was administered. Additionally, elevated levels of myocardial enzymes, including aspartate aminotransferase (AST), creatine kinase, and creatine kinase isoenzymes, suggested cardiac involvement, leading to the administration of sodium creatine phosphate for myocardial nutrition. Furthermore, the child developed tea-colored urine during hospitalization, considered to be caused by intravascular hemolysis, and was treated with alkalinization of the urine, although multiple renal function tests showed no abnormalities. Importantly, on the fourth day after admission, abdominal ultrasound revealed renal calculi, and cholecystolithiasis, as well as splenomegaly, considered complications of immune hemolysis. In addition, tests for antinuclear antibodies, ceruloplasmin, glucose-6-phosphate dehydrogenase activity, hepatitis viruses, and Epstein-Barr virus were all normal. The final diagnosis for the child was acute ceftriaxone-induced IHA, hemolytic crisis, myocardial injury, liver injury, renal calculi, and cholecystolithiasis. For a detailed overview of the key laboratory findings and special investigations undertaken during the diagnostic process, please refer to Table 1. After treatment, the child’s condition gradually improved, and he was successfully discharged after 12 days of hospitalization. One-month post-discharge, follow-up blood tests showed Hb levels of 153 g/L, an increase, and outpatient liver function tests indicated a return to normal. A 2-month follow-up abdominal ultrasound showed that the stones had disappeared. At 3 months, Hb levels had returned to the normal range of 129 g/L. Temporal changes in laboratory tests following ceftriaxone-induced immune hemolysis of the child was shown in Figure 1. Child’s progress from the onset of ceftriaxone-induced immune hemolysis to recovery, discharge, and follow-up was presented in Figure 2.

**TABLE 1:** Summary of laboratory investigations and special diagnostic procedures.

Investigations	Results	Reference range	Notes
Hemoglobin, g/L	42, further decreased to 21	112∼149	Highlights severe anemia
Blood glucose, mmol/L	15.4, gradually returning to normal	3.9∼6.1	Indicates stress hyperglycemia
Blood gas			Suggests metabolic acidosis and tissue hypoxia
pH	7.02	7.35∼7.45	
actual bicarbonate, mmol/L	5.5	18.0∼26.0	
base excess, mmol/L	−24.03	−3.0∼+3.0	
lactic acid, mmol/L	18	0.5∼2.2	
Blood biochemistry			
alanine transaminase, IU/L	a peak value of 844	7∼30	Indicates liver injury
Myocardial enzymes			Implies cardiac involvement
aspartate aminotransferase, IU/L	a peak value of 845	14∼44	
creatine kinase, IU/L	a peak value of 1054	50∼310	
creatine kinase isoenzymes, IU/L	a peak value of 99	0∼24	
Hemoglobin electrophoresis			Excludes hemoglobinopathies, such as sickle cell disease
hemoglobin A, %	97.20	95∼97.9	
hemoglobin F, %	0	0∼2.1	
hemoglobin A2, %	2.8	2.0∼3.5	
Peripheral blood smear	Normal	Normal	Helps in excluding various forms of hemolytic anemia associated with abnormal red blood cell morphologies, such as hereditary spherocytosis and sickle cell anemia
Glucose-6-phosphate dehydrogenase activity, U/L	2799	>1300	Excludes glucose-6-phosphate deficiency
Direct Antiglobulin Test	Positive	Negative	Indicates immune-mediated hemolysis
Antinuclear antibodies	Negative	Negative	Suggests a lower likelihood of systemic autoimmune disorders, such as systemic lupus erythematosus
Ceruloplasmin, g/L	0.24	0.15∼0.30	Excludes Wilson’s disease
Pathogen tests			Suggests no relevant pathogen infection and rule out Mycoplasma-induced immune hemolytic anemia
Epstein-Barr virus DNA	Negative	Negative	
*Mycoplasma pneumoniae* DNA	Negative	Negative	
Hepatitis viruses	Negative	Negative	
Coagulation function	No obvious abnormalities	Normal	suggesting the absence of coagulopathies such as disseminated intravascular coagulation, vitamin K deficiency
Renal function	No abnormalities	Normal	Rules out acute or chronic kidney diseases
Head and whole thoracoabdominal computed tomography	No abnormalities	Normal	Rules out internal bleeding
Chest X-ray	Enhanced pulmonary markings	Normal	Supports inflammation or infection in the bronchi
Ultrasound of abdomen	Renal calculi, cholecystolithiasis, and splenomegaly	Normal	Indicates complications of ceftriaxone-induced immune hemolytic anemia

## Discussion

This article reports on a previously healthy 3-year-old boy who experienced a severe immune hemolytic reaction and hemolytic crisis after continuous intravenous infusion of ceftriaxone. Fortunately, when the child suddenly turned pale, the attending physician quickly recognized the potential for a drug reaction to ceftriaxone and promptly discontinued its use, averting potentially grave consequences. This case serves as another warning about the use of ceftriaxone in children. Given the widespread use of ceftriaxone in pediatrics, it is crucial for pediatricians to remain vigilant about its adverse reactions. Timely cessation of ceftriaxone is key to achieving a favorable outcome once adverse reactions occur.

Adverse drug reactions caused by ceftriaxone are quite common in children, among which acute immune hemolytic reactions are one of the more severe adverse effects (Vehapoğlu et al., 2016; Leicht et al., 2018). Compared to adults, hemolytic reactions caused by ceftriaxone tend to be more severe in children (Leicht et al., 2018). There have been numerous cases reported that led to the death of children (Lu et al., 2018). When children also have hematologic disorders, such hemolytic reactions can be even more catastrophic. Bell et al. previously describes a case where an adolescent with Hb sickle cell disease (SCD) developed severe hemolytic anemia and hepatitis after receiving ceftriaxone therapy, leading to renal failure and multiple organ failure, ultimately resulting in death on the 19th day of hospitalization (Bell et al., 2005). A systematic review indicated that IHA following intravenous ceftriaxone led to the death of 11 children, all of whom had SCD as their primary condition. This suggests that having SCD is a high-risk factor for fatal outcomes in pediatric patients suffering from IHA induced by ceftriaxone (Zeng et al., 2020). In addition, life-threatening ceftriaxone-induced IHA has been reported in a child with Crohn’s disease (Mattis et al., 2004). Fortunately, our patient did not have any of these underlying conditions. Cases of ceftriaxone-induced immune hemolysis in pediatric patients under 5 years of age were displayed in Table 2.

**TABLE 2:** Cases of ceftriaxone-induced immune hemolysis in pediatric patients under 5 years of age.

Age (years)	Gender	Chief complaint	Hemoglobin (g/L) or hematocrit* (%)	Underlying condition	Outcome	References
3	Female	A sudden loss of consciousness	22	None	Favorable	Vehapoğlu et al. (2016)
4	Female	Apnea, bradycardia	N/A	Resected infant astrocytoma, panhypopituitarism, seizures	Favorable	Doratotaj et al. (2009)
5	Male	Back pain, seizures, fever, cough	N/A	Sickle cell disease	Favorable	Al-Hawsawi et al. (2010)
1.67	Male	Hypotonic; respiratory distress with bronchospasm; tonic-clonic seizure; shock	28	Congenital nephrotic syndrome	Favorable	Reis Boto et al. (2011)
2	Male	Loss of consciousness; hypotension; hypoxia	<0.10*	Sickle cell disease	Favorable	Goyal et al. (2011)
2	Male	Loss of consciousness; cardiac arrest	9	Sickle cell disease	Fatal	Bernini et al. (1995)
5	Male	Suddenly sat up and screamed with ear pain; blood was draining from his left ear, and he then stiffened, appeared pale, and became unresponsive	14	Juvenile ehronic myelocytic leukemia; neurofibromatosis; splenectomy	Fatal	Lascari and Amyot (1995)
3	Female	Pallor, hypotension, loss of consciousness	0.19*	Hypereosinophilic syndrome	Fatal	Scimeca et al. (1996)
5	Female	Seizure, decreased consciousness, pallor, dyspnea	N/A	None	Favorable	Citak et al. (2002)
5	Female	Oliguria, paleness and malaise	51	None	Favorable	Demirkaya et al. (2006)
0.67	Male	N/A	N/A	Pallor, restlessness, hepatosplenomegaly	Favorable	Candemir et al. (2006)
5	Female	Abdominal pain, vomiting, and hypotension	26	Disseminated low-grade glioma	Favorable	Pecker et al. (2016)

The acute IHA caused by ceftriaxone in children is a complex process, primarily mediated by immune mechanisms. Ceftriaxone-induced IHA can occur via two primary mechanisms: drug-independent and drug-dependent antibodies. Drug-independent antibodies behave like autoantibodies and cause autoimmune hemolytic anemia, while drug-dependent antibodies react with RBC only in the presence of the drug (Garratty, 2012). The most common mechanism that ceftriaxone—induced IHA is drug-dependent antibodies (Seltsam and Salama, 2000; Garratty, 2010). It has been reported that ceftriaxone can induce hemolytic anemia through three distinct mechanisms (Demirkaya et al., 2006). Initially, ceftriaxone functions as a hapten, binding to the RBC membrane and triggering the production of high-titer antidrug antibodies. These antibodies attach to the RBC membrane, leading to a positive DAT and subsequent hemolysis (Demirkaya et al., 2006). Additionally, it is suggested that the nonspecific absorption of ceftriaxone onto the RBC membrane, resulting in membrane modification, represents another mechanism contributing to immune-mediated hemolysis. DAT is also positive in this situation. However, this does not result in hemolytic anemia every time (Wells et al., 1991; Garratty, 2009). The final mechanism for ceftriaxone-induced hemolytic anemia is attributed to the immune complex mechanism, wherein preformed immune complexes of ceftriaxone and antibodies bind to RBC membranes, activating complement and leading to severe intravascular hemolysis (Arndt et al., 1999). These mechanisms are particularly significant in patients with existing hematologic disorders like SCD, where the RBC are already prone to hemolysis (Neuman et al., 2014), which help explain as previously mentioned why having SCD is a high-risk factor for fatal outcomes in pediatric patients suffering from IHA induced by ceftriaxone. In our case, the patient’s Hb levels were dramatically reduced to 42 g/L, further declining to 21 g/L after the withdrawal of ceftriaxone. Additionally, the RBC count decreased significantly to 0.91 × 10^12^/L, eventually reaching 0.31 × 10^12^/L, indicative of severe erythrocyte destruction. Furthermore, the DAT was positive. Therefore, our case’s diagnosis is more inclined towards ceftriaxone-induced IHA. Moreover, drug-dependent antibodies can also target blood platelet, leading to thrombocytopenia (Jacquot et al., 2013; Saha and Mitra, 2023). The binding sites of the drug-dependent antibodies could be localized to GPIIb/IIIa and GPIb/IX in platelet (Grossjohann et al., 2004). In our case, it was observed that the child mainly experienced a reduction in Hb levels, while platelet counts did not decrease during the course of the illness. This suggests that the ceftriaxone primarily caused immune hemolysis involving RBC in the present case, without affecting the platelet. Of note, in addition to ceftriaxone itself can induce IHA, its trace metabolites or degradation product can also induce severe and even life - threatening IHA [(Seltsam and Salama, 2000; Meyer et al., 1999; Kim et al., 2002)]. These results suggest that when using ceftriaxone, attention must be paid to the potential for inducing immune hemolytic reactions, even after the cessation of ceftriaxone, the possibility of immune hemolysis should still be monitored.

Ceftriaxone-induced immune hemolytic reactions in children are more commonly observed in those who have previously been treated with ceftriaxone due to a process known as sensitization. The involvement mechanism can be as follows. First, it refers to immunological memory. The initial exposure to ceftriaxone can stimulate the immune system to produce antibodies against the drug or its complex with RBC. Once produced, memory B cells remain in the body, ready to produce antibodies more quickly and robustly upon re-exposure to the same drug (Demirkaya et al., 2006). Second, it refers to accelerated response. Upon re-exposure to ceftriaxone, the immune system can recognize the drug more quickly, leading to a faster and more intense production of antibodies against the drug-RBC complexes. This accelerated immune response can result in a more rapid and severe hemolytic reaction. Third, it refers to increased antibody affinity. With repeated exposures, the immune system can generate antibodies with higher affinity for the drug or drug-cell complex, leading to more efficient binding and destruction of RBC (Leicht et al., 2018). Fourth, it refers to complement system activation. The antibodies bound to the RBC can activate the complement system, leading to cell lysis and hemolysis (Arndt et al., 1999). Previous exposure may increase the efficiency of this process during subsequent exposures. This phenomenon underlines the importance of carefully considering the history of drug exposure in pediatric patients and the potential risks associated with re-administering drugs known to cause immune-mediated reactions. Medical history taking, careful monitoring for signs of hemolysis, and consideration of alternative antibiotics when appropriate are crucial steps in managing the risk of severe immune hemolytic reactions in children treated with ceftriaxone.

The immune hemolytic reaction caused by ceftriaxone is a serious condition that can manifest with a range of symptoms in children, including pallor, loss of consciousness, seizures, and hypotension (Scimeca et al., 1996; Goyal et al., 2011; Vehapoğlu et al., 2016). This reaction occurs due to the drug inducing the production of antibodies against RBC, leading to their destruction, known as hemolysis. This process can lead to acute anemia, causing symptoms like pallor and loss of consciousness due to reduced oxygen carrying capacity of the blood. Seizures and hypotension may result from the sudden drop in RBC disrupting normal physiological functions. The mechanism behind other symptoms like back pain, hypotonia (reduced muscle tone), hypoxia (low oxygen levels), and shock is related to the body’s response to the rapid destruction of RBC and the release of their contents into the bloodstream (Bernini et al., 1995; Al-Hawsawi et al., 2010; Goyal et al., 2011; Reis Boto et al., 2011). For example, back pain might be associated with the aggregation of cellular debris in the kidneys, while hypotonia and hypoxia can result from inadequate oxygenation of tissues due to reduced RBC count. Shock can occur as a severe systemic response to the sudden imbalance in blood components, leading to reduced blood flow and oxygen delivery to vital organs. In our case, the patient’s symptoms of pallor, limb stiffness, and unresponsiveness were primarily due to the extensive destruction of RBC and tissue hypoxia. At the same time, the body made a rapid stress response, with a significant increase in blood sugar and a marked increase in WBC. Therefore, it can be observed that both blood sugar and WBC levels significantly increased. With the transfusion of RBC and the improvement of the oxygen-carrying capacity of Hb, the metabolic acidosis improved, and the body’s stress response was also alleviated (Figure 1).

The severe complications of ceftriaxone-induced IHA include acute renal failure (Seltsam and Salama, 2000; Bell et al., 2005), shock (Mayer et al., 2015), multi-organ failure (Al-Hawsawi et al., 2010; Goyal et al., 2011) and sudden cardiac arrest (Bernini et al., 1995). Moreover, hemolytic crisis is a dangerous potential adverse event (Goyal et al., 2011). Hemolytic crisis is defined by a sudden exacerbation of anemia with reticulocytosis (Goyal et al., 2011). In our case, the child presented with a hemolytic crisis, evidenced by a precipitous decline in Hb levels followed by an increase in reticulocyte count. Although reticulocytes were not initially measured in the days following the onset of symptoms, a marked elevation to 7% (compared to the normal range of 0.5%–1.5%) was observed on the fifth day after the onset of ceftriaxone-induced IHA (Figure 1A). Furthermore, cholelithiasis and nephrolithiasis are frequently observed complications associated with adverse reactions to ceftriaxone. A comprehensive systematic review revealed that the aggregated incidence rate of cholelithiasis stands at 18.8%, whereas nephrolithiasis or kidney stones occur at a rate of approximately 1.4% (Zeng et al., 2020). In our case, the patient developed nephrolithiasis and gallstones 4 days post-ceftriaxone cessation, echoing findings in a seven-year-old from a previous study (de Moor et al., 1999). Due to its pharmacokinetic characteristics, particularly the extended elimination half-life, ceftriaxone is recognized as a lithogenic substance (Garnier et al., 2023). It can form crystals in the urine or bile, specifically as calcium ceftriaxone (Daudon et al., 2018). Morphological examinations using phase-contrast and crossed polarized light microscopy reveal that these ceftriaxone crystals typically possess a needle-like shape and birefringent nature, aggregating into starburst patterns or irregular plates measuring up to 200 µm (Chutipongtanate and Thongboonkerd, 2011). Although previous study showed that ceftriaxone was crystallized with free calcium in dose- and time-dependent manner, ceftriaxone at therapeutic levels could be crystallized with free calcium in the urine under physiologic condition (Chutipongtanate and Thongboonkerd, 2011). Therefore, it is not surprising that our case developed cholelithiasis and nephrolithiasis even with normal blood calcium levels. Interestingly, ceftriaxone-induced stones are a type of pseudolithiasis (Biner et al., 2006). A prospective study in 284 children indicated an increased risk of kidney stone formation in children treated with ceftriaxone, with a 1.4% incidence rate of nephrolithiasis. However, stones passed spontaneously in all affected patients (Mohkam et al., 2007). In our case, a follow-up abdominal ultrasound 2 months later revealed that both gallbladder and kidney stones had disappeared, indicating that the stones had spontaneously passed.

Another significant feature of our case was the occurrence of drug-induced liver injury (DILI). According to the European Association for the Study of the Liver (EASL) guideline, drug-induced liver injury (DILI) case definitions include (EASL Clinical Practice GuidelinesClinical Practice Guideline Panel: Chair:Panel membersEASL Governing Board representative:, 2019): 1. ALT levels ≥5 times the upper limit of normal (ULN); 2. ALP levels ≥2 times ULN (excluding bone pathology related increases), especially with raised GGT; 3. ALT levels ≥3 times ULN with concurrent total bilirubin (TBL) > 2 times ULN. In our case, the ALT was up to 844 IU/L (normal range 7 ∼ 30 IU/L), GGT was 261 IU/L (normal range 5 ∼ 19 IU/L) (Figure 1B). Furthermore, other potential causes of infectious and autoimmune hepatitis were ruled out, as antinuclear antibodies were negative, and tests for Epstein-Barr virus infection, Wilson’s disease (ceruloplasmin), and hepatitis viruses all returned negative results. Therefore, it is no doubt that the patient was complicated with DILI after ceftriaxone-induced IHA. It is noteworthy that the transaminase levels peaked following the formation of gallstones. In a recent case reported by Castellazzi et al. showed that a 5-year-old child exhibited ceftriaxone-induced acute cholestatic hepatitis with marked elevations in ALT and GGT levels (Castellazzi et al., 2022). Bile acids trigger a hepatic inflammatory response, causing cholestatic liver injury (Zhang et al., 2023b). Therefore, in our case of ceftriaxone-induced liver injury in children, the mechanism causing liver damage may involves, firstly, acute hemolysis leading to a significant decrease in Hb and a consequent reduction in oxygen-carrying capacity, causing hypoxic liver injury (Birrer et al., 2007). Secondly, the formation of ceftriaxone-dependent antibodies might also directly damage liver cells by immune mechanism (Bell et al., 2005). Lastly, the formation of gallstones leads to bile stasis, further causing liver damage (Trauner et al., 2008). Fortunately, due to the liver’s exceptional regenerative and repair abilities (Fausto, 2000), along with hepatoprotective treatment with compound glycyrrhizin (Cao et al., 2006; Li et al., 2019; Chen et al., 2022), the child’s liver function normalized approximately 1 month following the removal of these harmful factors and subsequent follow-up.

For ceftriaxone-induced IHA, the most primary and crucial treatment is the timely withdrawal or discontinuation of ceftriaxone (Northrop and Agarwal, 2015; Zeng et al., 2020). As ceftriaxone-induced IHA is an immune response, avoid antigen exposure is critical (Parkin and Cohen, 2001). In our case, despite the severe immune hemolysis following ceftriaxone administration, the outcome was favorable, closely related to our timely identification and discontinuation of ceftriaxone. When we ceased ceftriaxone administration, the Hb level was 46 g/L, but it continued to decline, reaching a nadir of 21 g/L. Had we not promptly recognized the adverse reaction to ceftriaxone, the Hb could have deteriorated further, putting the child at significant risk. This is especially concerning given that reported fatal cases had Hb levels as low as 9.2 ∼ 14 g/L (Lu et al., 2018; Lascari and Amyot, 1995). Critically low Hb concentrations may be associated with an increased risk of mortality (Lascari and Amyot, 1995; Jung et al., 2019). Our case reiterates the importance of vigilance for atypical reactions, such as pallor or altered mental status, in individuals with prior ceftriaxone exposure. It accentuates the necessity of recognizing and promptly discontinuing ceftriaxone in suspected adverse reactions, a step that can be lifesaving. Alongside ceasing ceftriaxone, RBC transfusion is also crucial as it can correct anemia and improve oxygen-carrying capacity of the blood, thereby ameliorating organ and tissue hypoxia (Boggs et al., 2011). In our case, after transfusion of one unit of blood type O, Rh-positive concentrated RBC, the condition improved rapidly. Furthermore, administering IVIG may be necessary to mitigate further hemolysis. Vehapoğlu et al. successfully treated a 3-year-old girl’s ceftriaxone-induced severe IHA with IVIG, where her Hb had critically dropped to 22 g/L (Vehapoğlu et al., 2016). Our case is also a successful example of using IVIG to treat ceftriaxone-induced IHA. Yet, the specific role of IVIG in this success is not definitively clear. Unfortunately, a 5-year-old boy with ceftriaxone-induced IHA, whose Hb dropped to 9.2 g/L, succumbed despite receiving high-dose IVIG (Lu et al., 2018). Experts caution that IVIG treatment for drug-induced immune hemolysis should be used carefully because therapeutic IVIG can also cause acute hemolysis related to the passive transfer of antibodies, for example, to ABO or Rh antigens (Hill et al., 2017). Hence, the use of IVIG for ceftriaxone-induced IHA warrants further validation through rigorous evidence-based medicine. Additionally, corticosteroids are advocated as a therapeutic option for various AIHA, like systemic lupus erythematosus, owing to their potent anti-inflammatory and immunosuppressive properties. Reversely, the benefit of corticosteroids for drug-induced IHA is unclear (Hill et al., 2017). However, there are case reports of successful treatment with corticosteroids (Citak et al., 2002; Mulkens et al., 2015). Given the severe hemolysis in our case, we employed corticosteroids to mitigate further hemolytic activity. Fortunately, through a combination of medication cessation, transfusion, IVIG and corticosteroids administration, oxygen therapy, and close vital signs monitoring, the child successfully recovered and was discharged.

It is worth noting that while adverse reactions induced by ceftriaxone are being considered, some therapeutic measures might also contribute to the occurrence of certain symptoms. Sodium bicarbonate, commonly used to correct metabolic acidosis, has a dual effect on kidney stones. On one hand, it can alter urinary pH and potentially affect the solubility of certain minerals, which may increase the risk of renal calculi formation (Daudon et al., 2018). On the other hand, it can alleviate uric acid nephrolithiasis by alkalinizing the urine, enhancing the dissolution of uric acid stones and preventing their formation (Shekarriz and Stoller, 2002). Currently, there are no documented cases of sodium bicarbonate used for correcting metabolic acidosis leading to renal calculi in children. In our case, exposure to ceftriaxone and sodium bicarbonate subsequently led to the development of renal calculi. We speculate that the formation of renal calculi in our case was primarily induced by ceftriaxone. However, whether sodium bicarbonate played a role in this process remains unclear and warrants further investigation. Furthermore, In addition to causing acute hemolysis (Hill et al., 2017), IVIG therapy has been linked to liver damage (Björkander et al., 1988; Jiang et al., 2023) and acute renal failure (Conti et al., 2023). These adverse effects may arise from various mechanisms such as direct nephrotoxicity, immune complex deposition, and changes in hemodynamics (Conti et al., 2023). In our case, IVIG was administered only once before discontinuation. We meticulously monitored the patient for common adverse reactions associated with IVIG, such as acute hemolysis and renal failure, but did not observe these outcomes. It remains uncertain whether IVIG contributed to the liver injury mechanism. Nevertheless, after hepatoprotective treatment, the child’s liver function returned to normal during the 1-month post-discharge follow-up. Moreover, another aspect that requires special attention is blood transfusion. The effectiveness of RBC transfusions in AIHA patients is contentious due to concerns about heightened transfusion reaction risks. However, Park et al. showed that RBC transfusions in patients with AIHA are both effective and safe, not leading to an increased risk of hemolysis (Park et al., 2015). Additionally, it is advocated as a supportive treatment in cases of life-threatening anemia (Brodsky, 2019). Consistent with Park’s findings (Park et al., 2015), our observations also indicate that ceftriaxone-induced severe IHA did not lead to severe transfusion reactions.

Our case report has limitations, particularly in the diagnostic evaluation due to the absence of advanced serological tests for ceftriaxone-dependent antibodies, which could have provided more definitive evidence of the drug’s role in hemolysis. Additionally, while the comprehensive treatment, including IVIG, was effective, the broader application of these therapies necessitates further validation through evidence-based medicine to confirm their efficacy.

## Conclusion

In children, ceftriaxone infusion may trigger severe, potentially fatal, hemolytic reactions. Pediatricians must promptly recognize symptoms such as pallor, limb stiffness, and unresponsiveness, indicative of ceftriaxone-induced severe IHA, and immediately discontinue the drug. Effective management includes timely blood transfusion, respiratory support, IVIG administration, and corticosteroids when necessary, along with rigorous vital signs monitoring. Continued vigilance is imperative, even after cessation of ceftriaxone, to promptly address any residual adverse effects.

## Data Availability

The original contributions presented in the study are included in the article/Supplementary Material, further inquiries can be directed to the corresponding author.
